# Kisspeptins Regulating Fertility: Potential Future Therapeutic Approach in Infertility Treatment

**DOI:** 10.3390/jcm14103284

**Published:** 2025-05-08

**Authors:** Sonia Kotanidou, Nikos Nikolettos, Nektaria Kritsotaki, Panagiotis Tsikouras, Angeliki Tiptiri-Kourpeti, Konstantinos Nikolettos

**Affiliations:** 1 Department of Obstetrics and Gynecology, Democritus University of Thrace, 68100 Alexandroupolis, Greece; nnikolet@med.duth.gr (N.N.); kritsotaki.nk@gmail.com (N.K.); tsikouraspanagiotis@gmail.com (P.T.); 2 Genesis Athens-Thrace Medically Assisted Reproduction Unit, Apalos, 68100 Alexandroupolis, Greece; mbg_tiptiri@yahoo.gr; 3 Department of Gynaecological Oncology, Maidstone and Turnbridge Wells NHS Trust, Maidstone ME14 1FZ, UK; k.nikolettos@yahoo.gr

**Keywords:** kisspeptins, GnRH, infertility, therapeutic agents

## Abstract

Kisspeptins play a crucial role in the normal functioning of the reproductive axis in both humans and animals by stimulating the secretion of gonadotropin-releasing hormone (GnRH) from the hypothalamus. Recent studies have investigated the association of kisspeptins to infertility of diverse causes and the therapeutic potential of kisspeptins in infertility. Exogenous administration of kisspeptins appears to hold significant promise in restoring fertility, with ongoing studies in their application in ovarian stimulation protocols and as cryoprotectants during vitrification. This review provides a comprehensive analysis of the role of kisspeptins in reproductive physiology and their potential as therapeutic agents for infertility, highlighting their advantages over conventional treatments and their future prospects in clinical practice.

## 1. Introduction

Infertility is defined as the inability to achieve pregnancy after one year of unprotected intercourse [1]. Worldwide, it presents in 8–12% of reproductive-age couples and can result from factors related to either the female and/or male partner [2]. The pathophysiology of some conditions leading to infertility appears to be associated with a group of peptides known as kisspeptins [3,4,5]. Kisspeptins are considered the most potent stimulators of gonadotropin-releasing hormone (GnRH) secretion from the hypothalamus and their action is essential for normal reproductive function in both humans and animals [6,7].

Recently, the clinical efficacy of kisspeptin administration in individuals facing infertility has been investigated. Exogenous administration of kisspeptins in infertile patients exhibiting reduced kisspeptin expression may restore their fertility [8,9,10]. Furthermore, studies investigate their use in ovarian stimulation protocols or as cryoprotectants during vitrification [11,12]. However, therapeutic protocols utilizing kisspeptins for individuals with infertility have not yet been established, nor has their application been integrated into IVF protocols, as their therapeutic use is currently in its experimental stage and is being investigated for safety and efficacy. The use of kisspeptins may become widespread in the future due to the advantages they offer compared to currently utilized agents [13,14].

The purpose of this review was to present a thorough overview of the involvement of kisspeptins in the reproductive axis, and the potential use of kisspeptin analogs as a therapeutic approach to infertility in the future.

## 2. Kisspeptins System

Kisspeptins (Kp) are a group of structurally similar peptides synthesized by the KISS1 gene in humans and the Kiss1 gene in non-human species, respectively [15]. Kisspeptins are formed through the proteolytic processing of 145 amino acids precursor, which is proteolyzed further to four biologically active peptides consisting of 54, 14, 13, and 10 amino acids (kisspeptin-54, -14, -13, -10) [15,16]. KISS1, and its derived products, were initially known as suppressors of metastasis in multiple malignancies, such as melanoma, breast cancer, ovarian carcinoma, pancreatic carcinoma, and papillary thyroid carcinoma [15,17,18,19].

Despite the identification of KISS1 products in 2001, the reproductive aspect of the kisspeptin system remained undiscovered until 2003, when two independent studies found an association between mutations of their receptor (Gpr54) and idiopathic hypogonadotropic hypogonadism (iHH) [3,20]. This discovery revealed the correlation of kisspeptin system with the hypothalamic–pituitary–gonadal (HPG) axis, along with the crucial function in puberty onset and fertility. Kisspeptins function as strong stimulators of gonadotropin-releasing hormone (GnRH) secretion by attaching to their receptor—KISS1R [21]. The receptor is common for all the biologically active forms of kisspeptins [22].

The location of kisspeptin synthesis varies among species, as does their expression. In mammals, kisspeptin production is conducted in the arcuate nucleus (ARC) and the preoptic area (POA), whereas in rodents, it occurs in the arcuate nucleus (ARC) and the anteroventral periventricular nucleus (AVPV) [21,23]. The ARC population is responsible for the pulsatile secretion of GnRH and gonadotropins, while the POA population is responsible for generating the preovulatory surge of LH in individuals [21,23,24]. The ARC contains greater KISS1 neurons count compared to POA in mammals [25].

It is important to note that gonadal steroids regulate GnRH secretion through the kisspeptin system [26]. In particular, it has been observed that testosterone and estrogen can reduce the expression of Kiss1 mRNA in the ARC, whereas it increases the expression of Kiss1 mRNA in the anteroventral periventricular nucleus (AVPV), especially at the preovulatory phase across various species [26].

## 3. KNDy Neurons

Except kisspeptins, within the ARC, another two distinct neuropeptides are co-expressed that have been strongly associated with the feedback regulation of GnRH in POA [27]. The neuropeptides neurokinin B (NKB) and dynorphin (Dyn), along with kisspeptins, collectively define a subpopulation of neurons in ARC referred to as KNDy neurons [27]. NKB has a stimulatory effect, while Dyn an inhibitory effect on KNDy neurons; both function as co-transmitters and auto-regulators of KNDy neurons and control the secretory activity of kisspeptins. When secreted, kisspeptins bind to KISS1R expressed on GnRH neurons of POA and stimulate the secretion of GnRH [22]. A study by Topaloglu et al. showed that mutations in the gene encoding the NKB receptor (TACR3) are associated with idiopathic hypogonadotropic hypogonadism and infertility, confirming the correlation of KNDy neurons with gonadotropin secretion [28].

## 4. Association of Kisspeptins to Infertility

A defect in the kisspeptin signaling pathway appears to be linked to infertility [3,4,5,29]. Infertility is diagnosed as a failure to conceive after a year of regular unprotected coitus [30] and affects around one in six individuals globally, as reported by the World Health Organization (WHO) [2]. The kisspeptin system plays a critical role in the gonadal axis and has been strongly implicated in numerous causes of infertility, including among others idiopathic hypogonadotropic hypogonadism (iHH), hyperprolactinemia, primary ovarian insufficiency (POI), and polycystic ovarian syndrome (PCOS) [3,4,5,14,29].

### 4.1. Idiopathic Hypogonadotropic Hypogonadism (iHH)

Mutations or deletion of the Kiss1r gene, which encodes the kisspeptin receptor, lead to iHH in mammals and rodents, as studied initially by Seminara et al. and de Roux et al. in 2003 [3,20]. Differences between mice with the Gpr54 gene and Kiss1r-/- knockout mice have been reported in several studies, including observations of azoospermia, reduced testosterone levels, and smaller testicular size in male rodents [3,20]. In female rodents, findings include smaller ovaries, predominantly containing primary and secondary follicles; lower levels of 17β-estradiol; and an absence of mammary gland enlargement [3,20]. Additionally, both sexes showed a significant reduction in FSH and LH levels compared to the control group. [31,32]. The haploinsufficient Kiss1r mice (Kiss1r+/-) presented with a substantial reduction in the expression of ovarian Kiss1r and expedited primary ovarian insufficiency (POI) [33].

Studies have demonstrated that kisspeptins are necessary for the onset of puberty and specifically for the expansion of GnRH neuron activity [34]. Exogenous administration of kisspeptins in studies induces the production and secretion of GnRH from the pituitary gland, resulting in activation of the reproductive axis [34]. On the other hand, suppression of GnRH secretion, resulting in delayed onset of puberty, was observed by the administration of a kisspeptin antagonist (peptide 234) to female rodents [35,36].

### 4.2. Hyperprolactinemia

Clinical presentation of hyperprolactinemia in women consist of oligomenorrhea/amenorrhea, anovulatory cycles, galactorrhea, and infertility [37]. Elevated PRL levels inhibit the pulsatile secretion of FSH and LH, resulting in hypogonadotropic hypogonadism mediated via the kisspeptins system [5]. This is supported by the observation that PRL receptors are expressed in a low density on GnRH neurons, whereas KISS1R present PRL receptors in a higher density [5].

The presence of PRL receptors on KISS1 neurons and the suppression of mRNA KISS1 expression due to the elevated levels of PRL indicate the direct inhibitory effect of PRL on KISS1 neurons [8]. The pivotal role of kisspeptins in the pathogenesis of hyperprolactinemia-induced infertility was highlighted in a study by Sonigo et al., which demonstrated that exogenous administration of kisspeptins effectively restores gonadotropin secretion [8,9].

Another study presented the reversal of hyperprolactinemia-induced amenorrhea in two premenopausal women by the exogenous administration of kisspeptin-10 (KP10) [38]. The treatment led to an increase in estradiol and gonadotropin levels, accompanied by follicular growth in the ovaries [38]. Τhis response to the exogenous administration of KΡ10 supports the view that higher PRL levels act by directly suppressing KISS1 neurons. KP10 has the potential for use as a treatment in women who do not respond to therapy with cabergoline (a dopamine agonist) or in women undergoing neuroleptic treatment who wish to conceive [38].

### 4.3. Primary Ovarian Insufficiency (POI)

Studies conducted in animal models have demonstrated the association between the kisspeptin signaling pathway in the gonads and primary ovarian insufficiency (POI) [33]. Mice with Kiss1r haploinsufficiency exhibited progressive loss of oocytes/follicles, decreased numbers of preantral follicles, and infertility [33]. Additionally, the ovarian tissue displayed an atrophic appearance, with no mature follicles or corpus luteum present during the lifespan of the mice, along with reduced expression of Kiss1r mRNA [33]. Hormonal replacement with gonadotropins in cases of Kiss1r haploinsufficiency, where follicular development and ovulation do not occur, is ineffective [33].

Kisspeptins and their receptor are expressed in the gonads of rodents and mammals, playing a crucial role in ovarian reserves, steroidogenesis, the onset of puberty, and follicular ovulation [39]. The expression of KISS1/KISS1R is generally increased in granulosa cells and theca cells and varies depending on the day of the cycle [33,40]. The expression of KISS1/KISS1R in women increases with follicular development, reaching peak levels during the pre-ovulatory phase due to the increased secretory action of gonadotropins [41,42]. Furthermore, the ovarian expression of kisspeptins prevents follicular atresia and contributes to ovulation during the reproductive years [39].

### 4.4. Polycystic Ovary Syndrome (PCOS)

Panidis et al. [4] were the first to report that females with PCOS and normal body mass index (BMI) have elevated plasma levels of kisspeptin. Multiple studies later confirmed that women with PCOS, independently of BMI, exhibited augmented kisspeptin levels [43,44,45]. However, the observation is not confirmed when the population sample is statistically small, due to the wide phenotypic spectrum of PCOS [46,47,48]. A recent study also observed a correlation between kisspeptin levels and the age of women with PCOS, but further investigation with more samples is required to confirm this correlation and establish its clinical significance [49].

In women with PCOS, elevated levels of kisspeptins lead to hyperfunction of the HPG axis, resulting in dysregulation of the menstrual cycle. As previously mentioned, kisspeptins are the strongest stimulators of GnRH secretion, which has a significant impact on the secretion of LH [6,7]. Therefore, it is expected that women with PCOS will simultaneously exhibit elevated LH levels, a finding that is not substantiated by studies due to insufficient data. Nyagolova et al. [50] and Jeon et al. [51] confirmed the elevated levels of kisspeptins in women with PCOS; however, they noted that these levels did not exhibit a positive linear correlation with LH levels. The explanation for this phenomenon is provided in the study by Katulski et al., who observed that the secretion of kisspeptins and LH occurs simultaneously only in women with PCOS and normal menstrual cycles [52]. In women with PCOS and oligomenorrhea, the secretion pulses are not synchronized, suggesting that, in addition to menstrual irregularities, there is a disturbance throughout the entire reproductive axis [52].

### 4.5. Endometriosis

Matrix metalloproteinases (MMPs) play a significant role in the development of endometriosis, as they are responsible for the degradation of extracellular matrix proteins, cell migration, and infiltration. Studies have shown increased activity of MMP-2 and MMP-9 in the endometrial tissue of women with endometriosis compared to that of women without endometriosis [53,54,55]. The action of metalloproteinases appears to be inhibited by kisspeptins [56,57]. An immunohistochemical study by Abdelkareem et al. [58] demonstrated that the endometrium of women with endometriosis exhibited lower levels of KISS1 and KISS1R compared to women without endometriosis. This finding supports the hypothesis regarding the involvement of kisspeptins in the pathophysiology of endometriosis, as their increased expression in the endometrium inhibits the activity of metalloproteinases and cellular infiltration.

The expression of the KISS1 gene in ectopic endometrial tissue is elevated compared to that in the endometrium [59]. This supports the antimetastatic action of kisspeptins and the implantation theory, which posits that reduced KISS1 expression in the endometrium allows for the migration and implantation of ectopic tissue [59]. In contrast, a study by Makri et al. [56] did not detect KISS1 expression in any of the 17 samples from women with endometriosis, which may be attributable to the different methodology used in the studies. Regarding serum kisspeptin levels, women with endometriosis exhibit higher levels compared to the control group [60,61].

Kisspeptins play a significant role in the inhibition of ectopic endometrial migration and implantation. The difference in the expression of KISS1/KISS1R in ectopically implanted endometrial tissue compared to the endometrium of women with and without endometriosis suggests their potential future use as biomarkers for early diagnosis of endometriosis.

### 4.6. Unexplained Infertility

A review of the literature identified two studies that compared KISS1 levels in infertile couples based on the causative factor. The categorization included male factor infertility, female factor infertility, and unexplained infertility. Women with unexplained infertility had lower levels of kisspeptins compared to women with infertility of other etiologies. The researchers hypothesize that the reduced levels of kisspeptins may be due to mutations in the KISS1 gene, which affect the HPG axis in women [62,63].

### 4.7. Functional Hypothalamic Amenorrhea (FHA)

Kisspeptin levels in women with FHA and normal body mass index (BMI) in follicular phase are presented lower than in healthy women [64]. Jayasena et al. conducted a study where women with functional hypothalamic amenorrhea received exogenous kisspeptins, exhibiting a fourfold increase in LH levels compared to healthy women during the follicular phase [65]. This finding aligns with a study in rodents with hypothalamic amenorrhea, where reduced Kiss1 expression in the hypothalamus was noted, along with increased expression of its receptor (Kiss1r) [66]. Therefore, hypothalamic amenorrhea appears to be due to a deficiency of kisspeptins in the hypothalamus, and treatment with exogenous kisspeptins restores normal hormone secretion from the HPG axis [65].

Eating disorders are a cause of hypothalamic amenorrhea, and studies have shown an association between kisspeptins and energy reserves [67]. Specifically, food deprivation in rats led to a reduction in KISS1, KISS1 mRNA, and GnRH levels, which was reversed with food intake. KISS1 and KISS1 mRNA are expressed in various brain regions, including those that regulate food intake, though kisspeptin administration has no direct effect on appetite [66]. To maintain energy balance, energy intake must be equal to or exceed energy expenditure. Chronic negative energy balance (intake lower than expenditure) leads to infertility, as observed in animal models [68].

Administration of KP54 (37 µg/kg) subcutaneously twice daily for two weeks was studied in five women with hypothalamic amenorrhea. After the first dose of KP54, gonadotropin levels significantly increased, but by the 14th day of KP54 administration, the increase was noticeably diminished [65]. This observation is attributed to desensitization of the hypothalamus with daily KP54 administration. Estrogen levels remained consistent, and ultrasound findings did not indicate folliculogenesis. Based on these findings, the possibility of long-term kisspeptin administration was considered as a potential treatment for hormone-dependent tumors, due to its ability to suppress sex steroid hormone secretion [69], and for metastatic tumors [70].

In 2010, Jayasena et al. investigated the response and the degree of desensitization in women with hypothalamic amenorrhea to KP54 (37 µg/kg) when administered subcutaneously, twice weekly, for 8 weeks [10]. In this case, women exhibited an increase in gonadotropins from the first dose, with a comparable increase observed by the 14th day. The response to KP54 and gonadotropin levels remained consistent after 8 weeks. KP54 administration twice weekly resulted in a lower degree of desensitization compared to twice-daily administration, but it did not restore menstruation in women with hypothalamic amenorrhea.

### 4.8. Male Factor Infertility

Although the central role of kisspeptins in the hypothalamus is well established, their peripheral role in the testes remains unclear. Several studies have confirmed the expression of KISS1/KISS1R and Kiss1/Kiss1r in the male reproductive system in both human and animal models [19,71]. Specifically, they are expressed in Sertoli cells, spermatocytes, spermatids, and spermatozoa, indicating a potential autocrine or paracrine role in spermatogenesis in animal models [72]. In humans, the presence of KISS1/KISS1R has been identified in the tail, head, and neck of spermatozoa, suggesting a possible direct role in male fertility [73].

Men with infertility issues have been found to exhibit reduced kisspeptin levels in serum, likely due to dysregulation of the HPG axis. Sperm quality does not demonstrate a correlation with serum kisspeptin levels, whereas a correlation is observed with kisspeptin levels in semen [74]. A large cohort study conducted in China observed that kisspeptin levels in semen were 60,000 times higher than in serum, with a positive correlation between semen quality and kisspeptin levels in semen [75]. Due to this positive correlation with total sperm count and motility, it has been suggested that kisspeptins may contribute to spermatogenesis. In fact, a study on a fish species demonstrated that subcutaneous administration of kisspeptins accelerated spermatogenesis as a result of stimulating GnRH secretion [76]. Although the action of kisspeptins appears to mediate through the HPG axis, the expression of KISS1/KISS1R in the testes cannot exclude a direct effect on the testes [77].

Leydig cells in the testes are stimulated by LH to produce testosterone. Various studies have evaluated testosterone production in the testes following exogenous administration of kisspeptins and, despite the fact that male gonads express the KISS1R receptor, steroidogenesis appears unaffected. In contrast, a recent study provided in vivo evidence of increased testosterone levels following exogenous kisspeptin administration, after prior treatment with a GnRH antagonist (Acyline) in primates to exclude pituitary influence [78]. The GnRH antagonist inhibits LH secretion from the pituitary, indicating a direct action of kisspeptin on the male gonads.

## 5. Therapeutic Use of Kisspeptins

The most commonly used forms of kisspeptins in studies are KP10 and KP54. Their clinical utility is limited by their short duration of action and the need for parenteral administration. The half-life of KP10 is approximately 4 min in humans, while KP54 has a longer half-life of around 28 min. Bolus infusion of KP54 stimulates gonadotropin secretion in healthy women during the follicular phase, an effect not observed with bolus infusion of KP10 [79]. Additionally, intravenous administration of KP54 leads to a higher increase in LH levels compared to KP10 in healthy men [80,81]. KP10, due to its shorter amino acid sequence, is less expensive to produce than KP54 but exhibits greater biological instability [82].

Numerous studies have been conducted aiming to explore the potential therapeutic use of kisspeptins in patients with infertility. The pivotal role of kisspeptins in the onset of puberty and ovulation has led to the consideration of stimulating the HPG axis by exogenous kisspeptin administration. Although many factors have the ability to stimulate the HPG axis, kisspeptins appear to have advantages over these alternatives. The ability of kisspeptins to directly stimulate GnRH secretion from the hypothalamus enables their use for assessing hypothalamic function. Patients with iHH show a weaker response to kisspeptin administration compared to healthy individuals, as confirmed by two separate studies. In one study, patients with congenital hypogonadotropic hypogonadism exhibited a weaker response to KP54 administration compared to healthy men, and in another study, patients with iHH also showed a reduced response to KP10 compared to the control group [80,83]. The hypothalamic function test with KP10/KP54 can be used for the differential diagnosis between hypogonadotropic hypogonadism and delayed puberty [84].

One therapeutic application of kisspeptins is in functional gonadal disorders such as hypothalamic amenorrhea, hyperprolactinemia, and obesity-related hypogonadism. These clinical conditions are associated with kisspeptin deficiency. Age-related hypogonadism also appears to respond to the therapeutic use of KP54 [85].

A second therapeutic indication for the administration of kisspeptins is the pulsatile release of GnRH that they induce. Subcutaneous bolus administration of KP54 in healthy women during the follicular phase causes pulsatile LH secretion [86]. It is possible that women suffering from infertility due to insufficient GnRH secretion may be able to restore pulsatile LH secretion through kisspeptin therapy, thereby regaining their fertility.

Finally, due to the important role that kisspeptins play in the process of ovulation, their potential use for inducing ovulation in women undergoing IVF cycles has been studied. This application is particularly promising for women at high risk for OHSS. For this reason, more studies are being conducted to assess the safety and efficacy of ovulation induction with kisspeptins, compared to the currently used ovulation stimulants.

The first study involving the administration of kisspeptins in humans was conducted to investigate their potential therapeutic ability in infertile individuals and the effects of chronic administration [69,87]. The results showed an increase in gonadotropin levels in short-term therapeutic protocols, while repeated doses led to the phenomenon of tachyphylaxis (desensitization), making their therapeutic use ambiguous [87]. Chronic administration of kisspeptins, whether continuous or in repeated doses, may lead to reduced gonadotropin and sex steroid hormone levels [87]. This phenomenon has been observed in humans and animal models, regardless of the route of administration (intravenous, subcutaneous) [87]. While desensitization is undesirable in women with infertility, chronic use to suppress the HPG axis may be used therapeutically in hormone-dependent tumors, such as prostate cancer [87].

### 5.1. Triggering Ovulation

Healthy women participated in a study that observed a dose-dependent increase in LH levels after administration of KP54 [88]. The LH surge varied depending on the day of the cycle when KP54 was administered [88]. Specifically, higher LH levels were observed pre-ovulatory, sparking interest in investigating its potential use as an ovulation trigger [88].

The first trial of KP54 for ovulation induction was conducted in 2014 in women undergoing IVF [89]. The LH peak reached its maximum approximately 5 h after KP54 administration and lasted for 12–15 h, with most women having at least one mature oocyte retrieved [89]. This study demonstrated the effective use of KP54 for triggering ovulation [89].

LH levels after KP54 administration rise to levels observed in a natural mid-cycle LH surge (approximately 45 IU/L and 56.5 IU/L, respectively) [82,89], in contrast to the very high levels observed after GnRH agonist stimulation (approximately 140 IU/L) [90]. Notably, although KP54 induces a smaller increase in LH than the GnRH agonist, it achieves the same result in oocyte maturation [90]. This is attributed to the direct action of kisspeptins on KISS1R, which are expressed in the ovaries [39,91].

Due to the shorter duration of the LH surge following KP54 administration [92], a study was conducted in which a second dose of KP54 was administered 10 h after the first dose to examine whether a longer-lasting LH surge could be achieved. It was found that the group receiving two doses of KP54 had more mature oocytes retrieved compared to the group receiving a single dose [93].

Recently, MVT-602 (also known as TAK-448), a kisspeptin receptor agonist derived from the modification of KP10, has been studied. It exhibits a stronger pharmacodynamic effect than natural KP54 and increases LH levels, with a prolonged duration of action (21–22 h vs. 4.7 h, respectively) [13].

In 2024, a study including two randomized clinical trials was published investigating the endocrine profile of MVT-602 in healthy premenopausal women in follicular phase with and without ovarian stimulation [94]. The trials were placebo-controlled, parallel-group, and dose-ranging, showing that the LH surge induced by MVT-602 more accurately reflects the physiological midcycle LH surge that any current trigger agents. More precisely, intermediate dose of MVT-602 (1.0mg) produced the highest levels of LH concentrations with longer duration in comparison to native KP54, despite having similar elimination of half-lives, closely resembling the triphasic pattern of the physiological midcycle LH surge.

### 5.2. Ovarian Hyperstimulation Syndrome (OHSS)

A randomized controlled trial (RCT) investigated the possibility of preventing OHSS in women at high risk for the syndrome during ovulation induction with KP54 [92,95]. No women exhibited symptoms of moderate or severe OHSS, even in the study where a second dose of KP54 was administered [92,93]. The use of KP54 for ovulation induction is associated with a 33.6-fold reduced risk of severe OHSS compared to induction with rhCG [95]. Kisspeptin’s ability to prevent the onset of OHSS seems to be due to its short duration of action, and it is hypothesized that its direct action on ovarian KISS1R inhibits VEGF secretion, thereby reducing the incidence of OHSS [70,92].

In a retrospective study conducted in the United Kingdom, data were presented on OHSS cases in high-risk women following ovulation induction with rhCG, GnRH agonist, and kisspeptins [96]. OHSS was more significantly associated with the use of rhCG and GnRH agonist as ovulation triggers, in comparison to kisspeptin [96]. Additionally, the symptoms of OHSS caused by kisspeptin administration were milder than those caused by the other agents [96]. Finally, ovarian volume increased twentyfold compared to pre-induction volume in cases induced with synthetic hCG, eightfold with GnRH agonist, and fivefold with kisspeptin [96].

### 5.3. In Vitro Maturation (IVM)

For oocyte maturation, various growth factors are essential, such as GDF9, BMP15, and Kit, which are responsible for folliculogenesis, ovulation, and luteinization of the follicle [97]. The administration of KP10 has been shown to increase the expression of these growth factors. Studies have been conducted on the IVM of oocytes with KP10 administration in animal models but not yet in human oocytes [90,97].

In a recent study, the action of MVT-602, an agonist of the KISS1R receptor, was examined in cell lines, and signaling similar to that of KP54 in humans was observed [13]. There are future prospects for the use of KP54 and MVT-602 in the IVM of human oocytes but more studies are needed to establish safety of usage [13].

### 5.4. Cryopreservation

In recent years, the use of kisspeptins as antioxidant agents has been studied, as well as their effect on the expression of factors related to normal ovarian development. A study observed that the number of primary and secondary follicles in the group that underwent vitrification of ovarian tissue with kisspeptins was greater than that found in the group vitrified with other cryoprotective agents [11].

Furthermore, the level of apoptosis, the total antioxidant capacity (TAC) of the plasma, and the levels of superoxide dismutase (SOD) were examined in a further study by the same research group [12]. It was found that tissues that underwent vitrification with kisspeptins had the level of cell apoptosis reduced, while TAC and SOD were increased compared to the control group [12].

Various growth factors from the TGF-β family, specifically GDF9 and BMP15, are involved in paracrine signaling between the oocyte and the surrounding granulosa cells and play a significant role in oocyte maturation [98,99]. Several studies in the literature have investigated the expression of these factors in the ovaries of experimental animals and presented data showing reduced expression of GDF9 and BMP15 after cryopreservation and subsequent warming [98,99]. In a later study comparing classical vitrification with vitrification using kisspeptins, increased expression of GDF9 and BMP15 was observed in the vitrified samples with kisspeptins compared to the control group [97].

Based on studies in the literature, it appears that cryopreservation using the vitrification method and the use of kisspeptins as cryoprotectants may reduce the negative effects of cryopreservation by decreasing the generation of oxidative stress and apoptotic processes in the cell [11,12].

## 6. Conclusions

The literature review demonstrates the crucial role of kisspeptins in the onset of puberty, regulation of ovarian reserve, steroidogenesis, and ovulation. Mutations in the gene encoding kisspeptins or their receptor led to infertility. The kisspeptin system with the HPG axis in females has been elucidated, while their precise contribution to the male reproductive system remains unclear [22,74]. Further research is needed to clarify their role in steroidogenesis and their effect on male sperm quality [74].

The future use of kisspeptins may support differential diagnosis of delayed puberty [86], as well as offer treatment options for reversible infertility linked to their signaling pathways. For example, cases of central etiology infertility appear to respond to the administration of kisspeptin agonists, activating the HPG axis [65]. Although the physiology and pharmacokinetics of kisspeptins have been studied in recent years, the necessary therapeutic dosage and appropriate administration frequency are being tested in experimental studies and have not been established yet [65].

Additionally, kisspeptins are expressed in multiple tissues, including the endometrium. In women with endometriosis, the expression of the KISS1 gene in the endometrium is lower compared to that in ectopically implanted tissue [59]. However, one study disagreed with this observation, as KISS1 expression was not detected in any samples from women with or without endometriosis, likely due to methodological differences between the studies [56].

The hormonal response to the exogenous administration of kisspeptins in infertile women resembles the response observed in fertile women, due to endogenous kisspeptins and their signaling [90]. The administration of KP54 during the ovarian phase induces the pulsatile secretion of GnRH, resulting in an LH peak that reaches levels comparable to the mid-cycle peak seen in women with normal menstruation and ovulation [86,89]. Cases of infertility resulting from dysfunction in the kisspeptin system may potentially be treated in the future with kisspeptin analogs, which are considered safe for administration, and their use is being studied in central etiology infertility [80].

However, chronic administration of kisspeptins, as reported by Jayasena et al. [65], leads to desensitization of the axis, thus necessitating further research to determine therapeutic dosages. While desensitization of the gonadal axis is not desirable in infertility contexts, it may be therapeutic in hormone-dependent pathological entities or metastatic tumors, as it inhibits the production and secretion of sex steroid hormones [69,70].

Kisspeptins have potential applications in inducing ovulation, cryopreservation, and IVM of oocytes during IVF, as mentioned above [14]. The primary advantage of kisspeptin analogs is their short action on LH receptors, making them safer for inducing ovulation compared to rhCG, which is currently used [96]. Studies suggest that replacing rhCG with kisspeptins could reduce the incidence of OHSS [95]. However, their use remains experimental, with phase 1–2 trials currently underway examining the safety and efficacy of kisspeptin administration.

Considering the potential advantages of therapies including kisspeptins, it is important to study the benefits over current treatment options. Kisspeptins seem to stimulate a more physiological release of gonadotropins due to the endogenous pituitary release of GnRH they exert. As a result, kisspeptins may induce a more natural pattern of hormonal secretion rather than exogenous administration, reducing the possibilities of OHSS as revealed by clinical trials so far [100].

In conclusion, elucidating the action of kisspeptins provides a novel approach to regulating the HPG axis using kisspeptin analogs. Research conducted so far indicates that their administration is safe; however, further studies are necessary to determine their safety, side effects, and long-term effects in humans.

## Data Availability

This is a review, and all the data are published and cited in the text.
